# Regulation of Store-Operated Ca^2+^ Entry by Septins

**DOI:** 10.3389/fcell.2016.00142

**Published:** 2016-12-15

**Authors:** Bipan K. Deb, Gaiti Hasan

**Affiliations:** National Centre for Biological Sciences, Tata Institute of Fundamental ResearchBangalore, India

**Keywords:** STIM, Orai, flight, Endoplasmic reticulum, cytoskeletal proteins

## Abstract

The mechanism of store-operated Ca^2+^ entry (SOCE) brings extracellular Ca^2+^ into cells after depletion of intracellular Ca^2+^ stores. Regulation of Ca^2+^ homeostasis by SOCE helps control various intracellular signaling functions in both non-excitable and excitable cells. Whereas essential components of the SOCE pathway are well characterized, molecular mechanisms underlying regulation of this pathway need investigation. A class of proteins recently demonstrated as regulating SOCE is septins. These are filament-forming GTPases that assemble into higher order structures. One of their most studied cellular functions is as a molecular scaffold that creates diffusion barriers in membranes for a variety of cellular processes. Septins regulate SOCE in mammalian non-excitable cells and in *Drosophila* neurons. However, the molecular mechanism of SOCE-regulation by septins and the contribution of different subgroups of septins to SOCE-regulation remain to be understood. The regulation of SOCE is relevant in multiple cellular contexts as well as in diseases, such as the Severe Combined Immunodeficiency (SCID) syndrome and neurodegenerative syndromes like Alzheimer's, Spino-Cerebellar Ataxias and Parkinson's. Moreover, *Drosophila* neurons, where loss of SOCE leads to flight deficits, are a possible cellular template for understanding the molecular basis of neuronal deficits associated with loss of either the Inositol-1,4,5-trisphosphate receptor (IP_3_R1), a key activator of neuronal SOCE or the Endoplasmic reticulum resident Ca^2+^ sensor STIM1 (Stromal Interaction Molecule) in mouse. This perspective summarizes our current understanding of septins as regulators of SOCE and discusses the implications for mammalian neuronal function.

The calcium ion (Ca^2+^) performs multiple signaling functions to regulate a diverse range of cellular processes including fertilization, cell division, and apoptosis (Berridge et al., 2000; Clapham, 2007; Soboloff et al., 2012). Regulation of calcium signaling is thus of utmost importance in all cell types. Cytosolic Ca^2+^ concentrations are carefully maintained at levels of ~100–200 nM in most cell types either by extrusion of the excess Ca^2+^ into the extracellular space by ATP-driven pumps like the PMCA (Plasma membrane Ca^2+^ ATPase; Brini et al., 2013) or by sequestering excess Ca^2+^ into cellular organelles like the Endoplasmic Reticulum (ER), which functions as an intracellular Ca^2+^ store (Soboloff et al., 2012; Prakriya and Lewis, 2015). Ca^2+^ can enter the cell from the extracellular space in response to a variety of signals. For example, in T cells, activation of the T cell receptor on the cell surface activates intracellular signaling cascades and results in depletion of Ca^2+^ from the intracellular stores. This drop in intracellular store Ca^2+^ activates a mode of extracellular Ca^2+^ uptake called the store-operated Ca^2+^ entry (SOCE) (Putney et al., 2001; Putney, 2005; Prakriya and Lewis, 2015). The resulting increase in cytosolic Ca^2+^ stimulates translocation of the Nuclear Factor of Activated T-cells (NFAT) from the cytosol to the nucleus, where it turns on transcription of genes important for activated T-cell function (Lewis, 2001). SOCE is the primary mechanism for uptake of extracellular Ca^2+^ in non-excitable cells like T cells. Although discovered in immune cells, Ca^2+^ uptake by SOCE also operates in other non-excitable as well as in excitable cells, such as muscles (Pan et al., 2014) and neurons (Venkiteswaran and Hasan, 2009; Gruszczynska-Biegala et al., 2011; Hartmann et al., 2014).

## Store-operated Ca^2+^ entry (SOCE)

**Figure 1:**
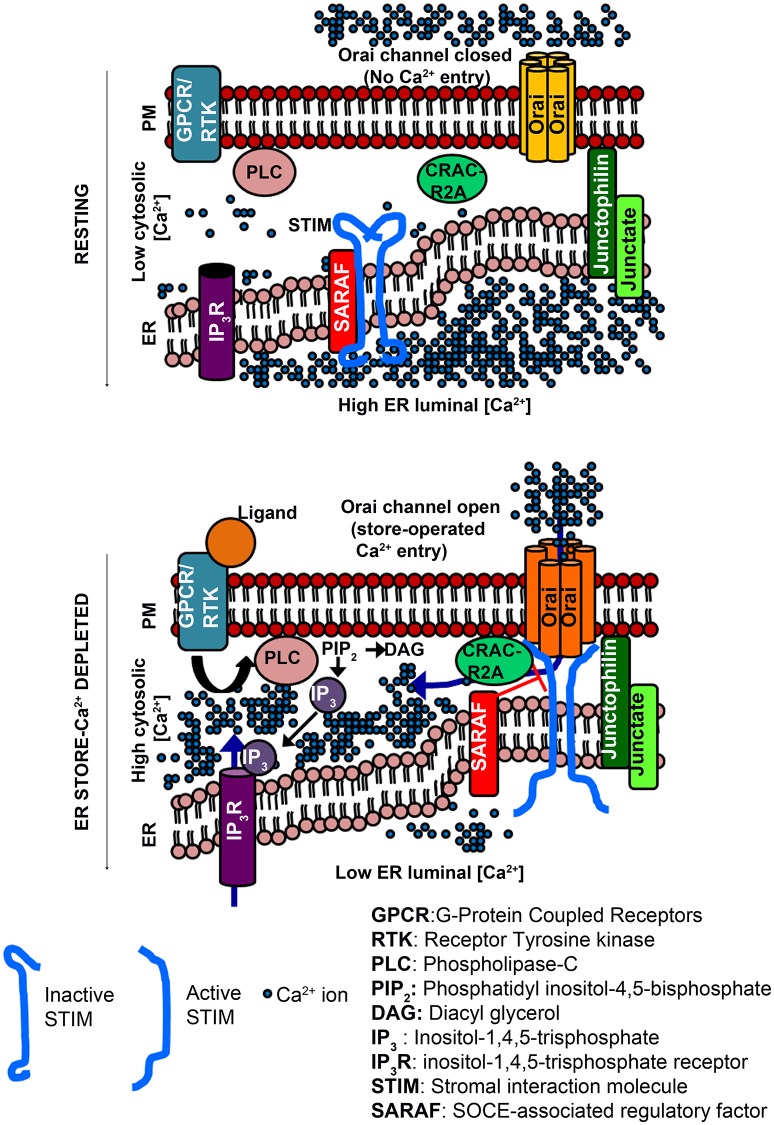
**Activation of Orai channels by STIM proteins during store-operated Ca^**2+**^ entry (Top)**. Cytosolic [Ca^2+^] is low compared to the ER luminal [Ca^2+^]. STIM proteins (inactive form) reside in regions of ER distal from the PM. Orai channels (closed) are distributed in the PM. ER-membrane resident Junctate and Junctophilin proteins (positive regulators of SOCE) help in pre-organizing ER-PM contact sites that facilitate STIM recruitment to the PM-proximal regions of the ER. **(Bottom)** Binding of an extracellular ligand to a PM-resident GPCR/RTK activates PLC and generates IP_3,_ which binds to ER-resident IP_3_R, and is followed by ER store-Ca^2+^ release through the IP_3_R. The lowering of ER luminal [Ca^2+^] activates STIM, which translocates to ER-PM regions where it binds to and opens Orai channels. CRACR2A is a positive regulator of SOCE that helps stabilize STIM/Orai complexes. SARAF is a negative regulator, which destabilizes STIM/Orai clusters to prevent excessive Ca^2+^ refilling. Hexameric Orai channels are depicted in accordance with the crystal structure of *Drosophila* Orai.

A range of extracellular signals can activate membrane-localized G-protein coupled receptors (GPCRs) and/or Receptor Tyrosine Kinases (RTKs) followed by Phospholipase C mediated enzymatic hydrolysis of Phosphatidyl inositol-4,5-bis phosphate (PIP_2_) and generation of the intracellular messenger inositol-1,4,5-trisphosphate (IP_3_). IP_3_ binds to the inositol-1,4,5-trisphosphate receptor (IP_3_R), which is primarily localized to the Endoplasmic Reticulum (ER) (Berridge, 2009). The IP_3_R is a ligand-gated Ca^2+^ channel and upon ligand binding releases Ca^2+^ from the ER to the cytosol along a favorable concentration gradient. The consequent drop in ER Ca^2+^ is sensed by ER-membrane localized Stromal Interaction Molecules (STIM) (Liou et al., 2005). The drop in ER Ca^2+^ results in release of Ca^2+^ from the luminal EF-hand domain of STIM, followed by oligomerization and translocation of STIM proteins to regions of the ER, which are in close proximity of the plasma membrane (PM), the ER-PM junctions (Zhang et al., 2005; Liou et al., 2007; Figure 1). STIM proteins recruited to the ER-PM junctions physically interact with the Ca^2+^-selective Orai channel located on the plasma membrane (Figure 1). In resting cells with replete Ca^2+^ stores, Orai normally remains closed. Upon ER store depletion and STIM translocation, STIM binds to Orai leading to channel opening and the entry of extracellular Ca^2+^, referred to as store-operated Ca^2+^ entry (Park et al., 2009; Figure 1). The resulting increase in Ca^2+^ levels in the cytosol serves as a signal for multiple Ca^2+^-dependent processes (Feske et al., 2001; Lewis, 2001; Somasundaram et al., 2014; Pathak et al., 2015). Cytosolic Ca^2+^ levels return to resting levels by the action of ATP-driven Ca^2+^ pumps on the ER (Sarco-Endoplasmic Reticulum Ca^2+^ ATPase or SERCA; Periasamy and Kalyanasundaram, 2007) and the PM (Plasma membrane Ca^2+^ ATPase, PMCA; Juška, 2010), which pump Ca^2+^ back from the cytosol to the ER lumen or the extracellular space, respectively (Soboloff et al., 2012; Prakriya and Lewis, 2015). Ca^2+^ from the cytosol can also be taken up along the concentration gradient by the Mitochondrial Ca^2+^ uniporter (MCU), a highly selective Ca^2+^ channel in the inner mitochondrial membrane (Kirichok et al., 2004; Baughman et al., 2011; De Stefani et al., 2011).

Several disease conditions arise as a consequence of either reduced or dysregulated STIM/Orai mediated SOCE. Mutations in genes encoding STIM1 and Orai1 reduce SOCE in T-cells leading to severe combined immunodeficiency (SCID) syndrome (Feske et al., 2006; Maus et al., 2015). STIM1 and Orai1 mediated SOCE also drives tumor metastasis in different kind of cancers (Yang et al., 2009; Chen et al., 2011, 2013). Congenital non-progressive myopathy in humans is associated with a loss of STIM1 and Orai1 (Stiber et al., 2008; McCarl et al., 2009) in agreement with findings *in vivo* and *in vitro* where loss of STIM1 resulted in muscle differentiation defects. The physiological relevance of SOCE in neurons is only just beginning to be understood. Knockdown of single genes encoding *Drosophila* STIM and Orai, *dSTIM* and *dOrai*, respectively, in neurons compromised flight initiation and maintenance (Venkiteswaran and Hasan, 2009). Defects in motor coordination were also observed upon knock out of STIM1 from cerebellar Purkinje neurons in mice (Hartmann et al., 2014). SOCE thus serves important signaling functions in a range of cell types by regulating cellular Ca^2+^ homeostasis.

## Septins regulate SOCE

Stromal Interaction Molecule (STIM) and Orai are the key molecular components of SOCE, but in addition other proteins like CRACR2A (CRAC regulator 2A; Srikanth et al., 2010) and Junctophilin-4 (Woo et al., 2016) modulate the strength and duration of the Ca^2+^ signal and subsequent downstream events. While CRACR2A facilitates STIM/Orai coupling after ER store-depletion (Srikanth et al., 2010), Junctate and Junctophilin help define ER-PM junctions suitable for STIM/Orai coupling (Srikanth et al., 2012; Woo et al., 2016). A genome wide screen in HeLa cells identified Septins as positive regulators of SOCE (Sharma et al., 2013). Septins are a class of filament forming GTPases that assemble into higher order structures. Primarily they act as diffusion barriers or molecular adaptors (Mostowy and Cossart, 2012). However, septin function can vary depending on the cell type as well as the context of a given cell type. For example, knockout of SEPT7 blocks cell division in fibroblasts but not in lymphocytes (Sellin et al., 2011; Menon et al., 2014). T-lymphocytes in contact with other cells do not require septins for division, whereas single T cells that are not in contact with other cells depend on septins for cell division (Mujal et al., 2016).

Based on their sequence homology, Septins can be classified into four subgroups- SEPT2, SEPT6, SEPT7, and SEPT3 (Kartmann and Roth, 2001; Kinoshita, 2003; Pan et al., 2007). Septins of different subgroups occupy distinct positions in a linear septin filament. Determination of the structure of a hexameric oligomer formed by human SEPT7, SEPT6, and SEPT2 revealed that members of the SEPT2 subgroup occupy the central position whereas SEPT7 occupies the terminal position in such a complex (Sirajuddin et al., 2007). Originally, it was shown that loss of SEPT2, SEPT4, and SEPT5 reduced SOCE significantly in Jurkat T-cells (Sharma et al., 2013). All three belong to the SEPT2 subgroup (Kartmann and Roth, 2001; Kinoshita, 2003; Pan et al., 2007).

**Figure 2:**
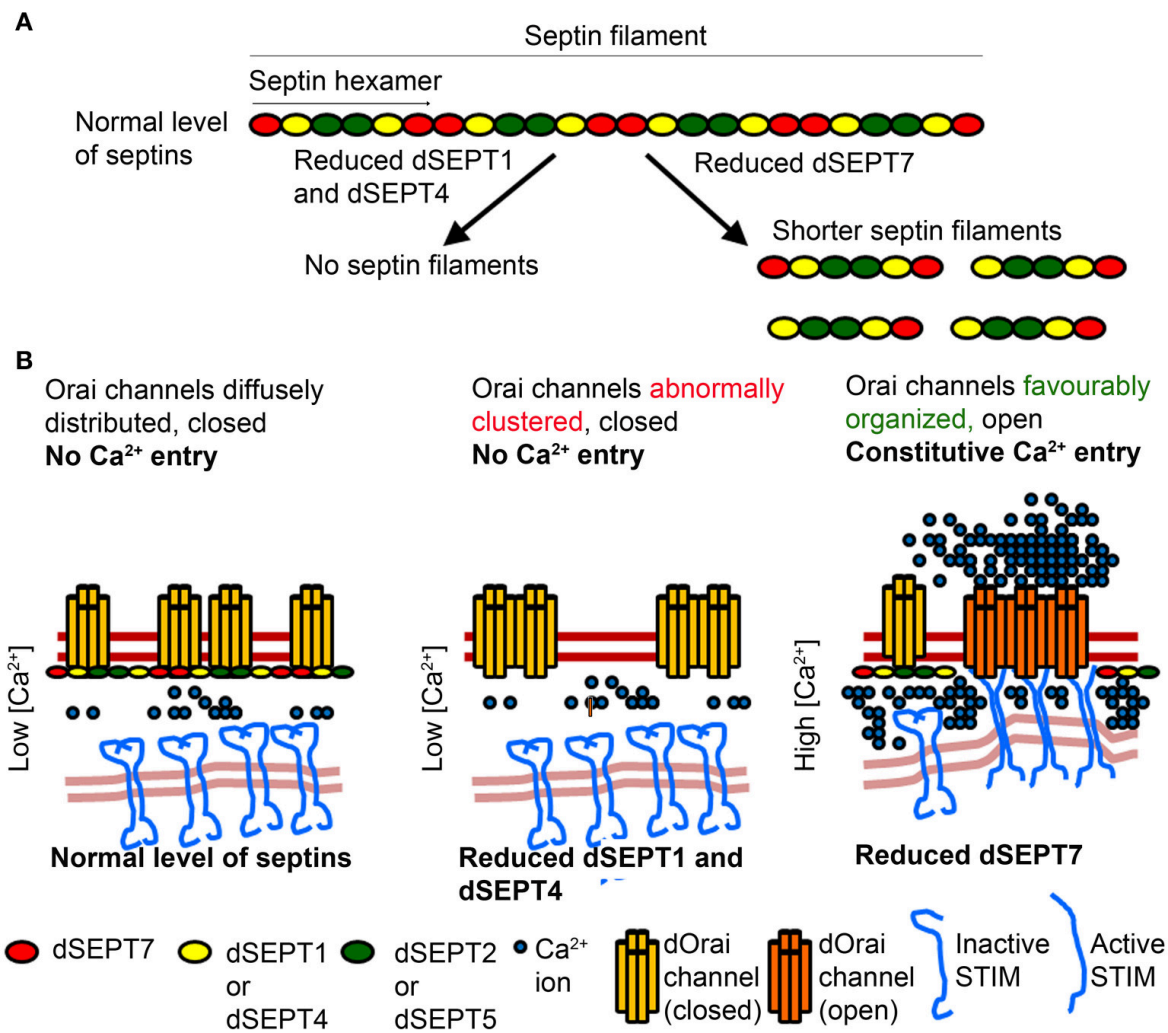
**Proposed mechanism by which Septins of the SEPT2 subgroup affect Septin filaments and Orai activation differently from SEPT7 (A)** Septin subunits belonging to different subgroups form hexameric complexes that are arranged end to end to form linear non-polar filaments. Reduction of the SEPT2 subgroup, dSEPT1 (or Sep1) and dSEPT4 (or Sep4), results in loss of septin filaments. Reduction of dSEPT7 (or Pnut) results in formation of shorter septin filaments. **(B)** In resting cells with normal level of Septin subunits, septin filaments help organize lipid domains in the PM, with closed Orai channels. Reduction of the SEPT2 subgroup leads to loss of septin filaments and favor's a closed conformation of Orai channels. Reduction of dSEPT7 results in shorter septin filaments, which facilitate STIM/Orai coupling and the distribution of Orai in lipid domains, where a constitutively open conformation is favored. Septin filament and Orai architecture depicted in the model are based on genetic and cellular studies but have not been demonstrated experimentally.

In addition to positive regulators of SOCE, given the necessity of tightly regulating cellular Ca^2+^ entry, not surprisingly, negative regulators of SOCE have also been identified (Feng et al., 2006; Palty et al., 2012). For example, SARAF (SOCE-associated Regulatory Factor) regulates SOCE by destabilizing STIM1/Orai1 complexes (Palty et al., 2012). Recent genetic and cellular experiments in *Drosophila* neurons demonstrated that reduced levels of dSEPT7 support store-independent Ca^2+^- entry through dOrai. Thus, SEPT7 functions as a negative regulator of the *Drosophila* Orai channel in neurons (Deb et al., 2016). The *Drosophila* genome contains five genes encoding septins (Neufeld and Rubin, 1994; Field et al., 1996; Adam et al., 2000) that have been classified into three subgroups based on their sequence homology with mammalian septins (Cao et al., 2007). dSEPT1 (or “Sep1”) and dSEPT4 (or “Sep4”) belong to the SEPT2 subgroup while dSEPT2 (or “Sep2) and dSEPT5 (or “Sep5”) belong to the SEPT6 subgroup of septins (Figure 2A). Similar to mammals, dSEPT7 is the only member of the SEPT7 subgroup in *Drosophila* (Cao et al., 2007). There are no representative members of the SEPT3 subgroup in *Drosophila*. An important question that arose from these findings is whether Septin subunits regulate SOCE differentially in *Drosophila* neurons as compared to mammalian T-cells.

## Septin subgroups and regulation of the orai channel

The role of septin subgroups and how they might independently modulate SOCE in mammalian cells remains to be addressed. Recent work in *Drosophila* neurons suggests a complex picture. Reduction of SOCE in neurons affects flight initiation and maintenance in *Drosophila* (Venkiteswaran and Hasan, 2009). Simultaneous knockdown of dSEPT1 and dSEPT4 in neurons reduced neuronal SOCE and resulted in flight deficits in *Drosophila*, indicating that the SEPT2 subgroup of septins, dSEPT1 and dSEPT4, function as positive regulators of SOCE in *Drosophila* flight circuit neurons (Deb et al., 2016). These observations are in agreement with studies in mammalian cells (Sharma et al., 2013), suggesting that the role of SEPT2 subgroup septins in SOCE is evolutionarily conserved across cell types. Septin 7 regulation of SOCE in *Drosophila* neurons appears more nuanced. Reduction of dSEPT7 had no significant effect on SOCE in *Drosophila* neurons. However, reduction of dSEPT7 in *Drosophila* neurons with mutations in the IP_3_R or with reduced levels of the Ca^2+^ sensor dSTIM, enhanced uptake of extracellular Ca^2+^ (Deb et al., 2016). This Ca^2+^ entry occurred without depletion of ER store Ca^2+^ and was abrogated by introducing a dominant negative form of dOrai, suggesting that reduction or loss of dSEPT7 activated dOrai constitutively in *Drosophila* neurons. In agreement with this, basal cytosolic Ca^2+^ levels were significantly higher in resting neurons with reduced or no dSEPT7 when compared to wild-type neurons. dSEPT7 also forms a hexameric complex with dSEPT2 and dSEPT1 similar to the mammalian SEPT7-SEPT6-SEPT2 complex (Field et al., 1996). An intriguing question that arises from Septin/SOCE findings in *Drosophila* neurons is how two subgroups of Septin subunits, viz., dSEPT1/4 and dSEPT7, both of which form part of the same complex, perform antagonistic rather than synergistic functions for Orai channel activation. Whereas, dSEPT7 acts as a negative regulator or “molecular brake” of the dOrai channel in *Drosophila* neurons, SEPT2 subgroup septins (dSEPT1/4) exhibit an essential role in SOCE activation. A possible explanation for these contrasting observations is that SEPT7 can function independently of other septin subunits, but existing data from mammalian cells do not support this idea (Sellin et al., 2011). Though an independent function for dSEPT7 cannot be ruled out, an alternate explanation for these apparently contradictory findings might lie in the assembly order of these septins during formation of septin complexes and the subsequent filament structure.

Septins of the SEPT2 subgroup nucleate septin complex formation. Knockdown of the SEPT2 subgroup destabilizes septin complexes in mammalian cells (Sellin et al., 2011). In *Drosophila* mutations in the GTPase domain of dSEPT1, which belongs to the SEPT2 subgroup, prevents stable septin complex formation whereas mutations that attenuate GTPase function of dSEPT7 do not affect it's ability to form a stable complex with wild-type dSEPT1 and dSEPT2 (Akhmetova et al., 2015). Mutant studies of genes encoding *Drosophila* SEPT6 subunits (*dSEPT2* and *dSEPT5*) suggest that similar to mammalian cells a SEPT6 class subunit is necessary for functional Septin complexes (O'Neill and Clark, 2016). In mammalian cells, reduction of SEPT7 affects the formation of complexes with septin hexamers but allows formation of heterodimeric and heterotetrameric complexes containing SEPT2 and SEPT6 (Sellin et al., 2011). We hypothesize that partial reduction of dSEPT7 leaves SEPT2-SEPT6 complexes intact and results in formation of smaller septin filaments, because filament elongation requires dSEPT7 at the two termini (Figures 2A,B). Septin filaments are known to help in the formation of lipid domains in the plasma membrane. For example, boundaries created by septin filaments prevent lateral diffusion of proteins between the membrane of mother and daughter cells in yeast (Barral et al., 2000; Faty et al., 2002). Thus, we speculate that in resting cells, septin filaments help in sequestering dOrai in lipid domains that are non-permissive to STIM/Orai interactions and Orai opening. Partial loss of dSEPT7 in resting cells results in shorter septin filaments and helps organize membrane lipids in a conformation that is permissive to STIM/Orai coupling and Orai opening (Figure 2B). Indeed, partial reduction of dSEPT7 resulted in constitutive activation of dOrai and increased clustering of dOrai in resting cells (Deb et al., 2016).

In mammalian cells, knockdown of SEPT2/4/5 resulted in abnormal clustering of Orai1 in resting conditions (Sharma et al., 2013). Septins bind phosphoinositides through a polybasic domain (Zhang et al., 1999; Bertin et al., 2010) and the polybasic domain of mammalian SEPT4 binds preferentially to PIP_2_ (Zhang et al., 1999). In cells with SEPT2/4/5 knockdown, diffuse and homogeneous distribution of Orai1 was replaced by Orai1 clusters that correlated with an altered arrangement of PIP_2_ in the PM (Sharma et al., 2013). These observations suggest that septin filaments help maintain PIP_2_ organization in the PM necessary for Orai activation after store-depletion. The abnormal Orai1 clusters are a likely cause for reduced STIM1/Orai1 coupling observed in cells with knockdown of SEPT2/4/5 after ER-store depletion. Because SEPT2/4/5 subunits are required to nucleate septin complex formation, we hypothesize that their reduction destabilizes septin filaments. Very likely, this affects Orai organization in resting cells, and upon SOCE stimulation negatively impacts both STIM1/Orai1 coupling and Orai1 opening (Figure 2B). Additional cellular studies and higher resolution analysis of Orai organization by electron microscopy in cells with knockdown of either SEPT7 or SEPT2/4/5 subunits should help elucidate the mechanisms by which different Septin subunits control Orai organization and channel opening.

## Septins and the ER-PM translocation of STIM

An essential step in regulation of SOCE is movement of the ER-Ca^2+^ sensor STIM to ER-PM junctions after store depletion, followed by its interaction with Orai, leading to channel opening and Ca^2+^ entry. Interestingly, septin filaments were detected both in the vicinity of the PM as well as at the ER in *Drosophila* neurons and reduction of dSEPT7 increased the intensity of dSTIM near the PM in resting neurons (Deb et al., 2016). A role for Septin regulation of STIM movement is also indicated in mammalian cells where knockdown of the SEPT2 subgroup of septins led to a delay in STIM recruitment to the ER-PM regions after ER-store depletion (Sharma et al., 2013). These data suggest that septins also help in the recruitment of STIM to the ER-PM junctions. Taken together these findings support the idea that septin filaments localized at the ER regulate STIM translocation and consequently its affect on Orai organization. In addition, at the PM, Septin filaments interact with PIP_2_ and probably function as diffusion barriers. In the case of dSEPT7 knockdown, partial loss of the diffusion barrier might allow Orai opening, whereas knockdown of the SEPT2 subgroup results in complete or near-complete loss of Septin filaments and consequently a complete loss of the diffusion barrier, which impacts Orai activation negatively.

Several questions that need addressing emerge from these observations. Firstly, the nature of Septin complexes formed in *Drosophila* neurons and their specific organization in the ER and PM regions before and during SOCE need to be understood. Secondly, a better molecular understanding of dOrai channel opening by reduction of dSEPT7 is necessary. The observation that Ca^2+^ entry through Orai upon dSEPT7 reduction remained unaffected by knockdown of dSTIM suggests that the Orai channel in neurons with reduced dSEPT7 may exist in a conformation, that allows Ca^2+^ entry in the absence of SOCE. Importantly, we do not know as yet the effect of complete loss of dSEPT7 on SOCE. If dSEPT7 null neurons lack Septin filaments, it is possible that this too will prevent Orai channel opening. Finally, at this stage it is not known how knockdown of dSEPT1/4 (SEPT2 subgroup in *Drosophila*) alters clustering and activation of dOrai and conversely if knockdown of SEPT7 in mammalian cells has similar effects on Orai mediated Ca^2+^ entry as seen after dSEPT7 reduction in *Drosophila* neurons.

The observation that reduction of dSEPT7, restored normal flight in flies with reduced IP_3_R or STIM function suggests that Septin 7 could be a target for alleviating conditions arising from reduced SOCE in neurons (Deb et al., 2016). Dysregulated Ca^2+^ signaling is linked to several neurodegenerative disorders (Egorova et al., 2015). If indeed, SEPT7 functions as a “molecular brake” on the Orai channel in mammalian neurons, it could be an important therapeutic target for diseases resulting from reduced intracellular Ca^2+^ signaling in neurons.

## Author contributions

All authors listed, have made substantial, direct and intellectual contribution to the work, and approved it for publication.

## Funding

This work was funded by a core grant from the National Centre for Biological Sciences, TIFR to GH and a Research Fellowship by the Council of Scientific and Industrial Research, Govt. of India to BD.

### Conflict of interest statement

The authors declare that the research was conducted in the absence of any commercial or financial relationships that could be construed as a potential conflict of interest.
